# Osteomorphological Description and Osteometric Variation of Long Bones in Ethiopian Indigenous Naked Neck Chickens in Quara District, Northwest Ethiopia

**DOI:** 10.1016/j.vas.2026.100880

**Published:** 2026-09-24

**Authors:** Zerihun Getie Wassie, Bemrew Admassu Mengistu, Gashaw Getaneh Dagnaw, Saleamlak Abebe Bihonegn, Abdo Megra Geda, Assaye Wollelie Fentie, Teketay Bayleyegn Derso, Kefale Ambachew Shiferaw, Abraham Belete Temesgen

**Affiliations:** a Department of Veterinary Biomedical Sciences, College of Veterinary Medicine and Animal Sciences, University of Gondar, P.O. Box: 196, Gondar, Ethiopia; b Department of Veterinary Pathobiology, College of Veterinary Medicine and Animal Sciences, University of Gondar, Gondar, Ethiopia; c School of Veterinary Medicine, College of Agriculture, Woldia University, Woldia, Ethiopia; d Department of the Donkey Sanctuary Project, College of Veterinary Medicine and Agriculture, Addis Ababa University, Bishoftu, Ethiopia

**Keywords:** Age group, *Gallus gallus domesticus*, Long bones, Naked Neck chicken, Osteometry, Sex

## Abstract

Osteomorphological and osteometric assessments provide valuable information on skeletal characteristics and biological variation in indigenous chicken populations, yet data on the long bones of Ethiopian Indigenous Naked Neck chickens remain limited. This study aimed to characterize osteomorphology and evaluate osteometric variation of selected long bones in Indigenous Naked Neck chickens from Quara District, Northwest Ethiopia. Sixteen clinically healthy chickens, comprising eight males and eight females, were allocated to two predefined age groups: 8-month-old younger chickens and 14-month-old older chickens. The humerus, radius, ulna, femur, tibia, and tarsometatarsus were examined morphologically and assessed using standardized osteometric procedures. Significant differences in bone weight and osteometric measurements were observed between the predefined age groups and sexes (*P* < 0.05). Older chickens exhibited greater bone weight and linear dimensions than younger chickens, while males showed larger measurements than females. Bone weight displayed greater relative variability than most linear measurements, as indicated by higher coefficients of variation. The osteomorphological features observed were consistent with established descriptions of avian skeletal anatomy. However, because individual body weight data were not available for inclusion as a covariate and potential age × sex interactions were not evaluated, the findings should be interpreted as observed group differences rather than independent effects of age or sex. These results provide baseline osteometric data for Indigenous Naked Neck chickens and serve as a reference for comparative anatomy and future studies of skeletal development and variation. Further studies involving larger and more diverse populations, individual body weight measurements, and factorial statistical approaches are warranted.

## Highlights

• First osteomorphological and osteometric dataset for Ethiopian Indigenous Naked Neck chickens.
• Osteometric measurements differed between predefined age groups and sexes.
• Older chickens and males generally exhibited larger long-bone measurements.
• Findings provide baseline data for comparative anatomy and skeletal biology studies.
• Future studies with larger sample sizes should evaluate age × sex interactions using factorial statistical models.

## 1 Introduction

Chickens (*Gallus gallus domesticus*) are widely distributed across diverse ecological environments and play an important role in traditional poultry production systems because of their adaptability and relatively low management requirements (Getu et al., 2014). Indigenous chicken populations are important genetic resources that contribute substantially to rural livelihoods, household nutrition, and food security, particularly in developing countries. Chickens possess a specialized skeletal system that provides structural support, protects internal organs, and facilitates locomotion (Chaurasia et al., 2018). The avian skeleton exhibits considerable morphological and osteometric variation, particularly within the appendicular skeleton, which can reflect differences associated with growth, sex, age, breed or population, and environmental adaptation (Khan et al., 2021; Doğan & Takcı, 2021). Long bones are particularly useful indicators of skeletal variation because their dimensions and morphology change during growth and may differ according to sex, age, genetic background, and functional demands. Their relatively well-defined anatomical landmarks also allow reliable morphological description and quantitative measurement (Kigir et al., 2022).

Beyond their mechanical role, avian skeletal structures function as important mineral reservoirs, particularly for calcium and phosphorus, which are essential for physiological processes such as eggshell formation in laying hens (Frey et al., 2001). Osteological studies provide foundational information for comparative anatomy, taxonomy, evolutionary biology, archaeology, and structural biology (Thomas et al., 2016). In chickens, osteometric measurements of long bones have been used to characterize skeletal development and variation among breeds and populations, with differences reported in parameters such as bone length, diameter, circumference, and weight (Mabelebele et al., 2017; Vitorović et al., 2009). Such measurements can therefore provide useful baseline information for comparing populations and evaluating skeletal variation among age groups and sexes. The long bones of the pectoral and pelvic limbs are particularly suitable for these investigations because of their consistent anatomical organization and clearly distinguishable landmarks (Abdulrahman & Yusuf, 2021).

Avian bones develop through both intramembranous and endochondral ossification and are structurally adapted to balance reduced skeletal weight with adequate mechanical strength (Tatara et al., 2005; Doğan & Takcı, 2021). The skeletal system is traditionally divided into axial and appendicular components, while individual bones are classified as long, short, flat, irregular, or sesamoid based on their morphology. At the microscopic level, cortical and trabecular bone structures contribute to skeletal integrity, locomotor efficiency, and mineral storage (Behera et al., 2021). Because long bones are directly involved in weight bearing and locomotion, their morphology and dimensions may also reflect functional adaptation to body size and mechanical loading. Consequently, osteomorphological and osteometric assessment of these bones can provide useful information for skeletal characterization and comparative anatomical studies. Osteometric parameters such as bone length, diameter, circumference, and weight provide essential baseline data for characterizing skeletal development and variation among chicken populations and breeds (Vitorović et al., 2009; Mabelebele et al., 2017).

Detailed morphological descriptions of long bones are also useful for skeletal identification, growth assessment, and breed or population characterization (Vitorović et al., 2009; Abdulrahman & Yusuf, 2021). Previous studies in chickens have demonstrated measurable differences in skeletal dimensions among breeds, populations, sexes, and developmental stages, supporting the use of long-bone measurements as indicators of biological and functional variation (Mabelebele et al., 2017). However, the extent and pattern of such variation may differ among genetic populations, emphasizing the need for population-specific baseline data (Khan et al., 2021).

Despite their economic and genetic importance, detailed information on the skeletal morphology and osteometry of Ethiopian indigenous chickens, particularly the Naked Neck ecotype, remains limited. Population-specific osteological data are essential for comparative anatomy, developmental studies, and the assessment of skeletal variation among different age groups and sexes. Therefore, this study aimed to characterize the gross osteomorphology and establish osteometric reference values for selected pectoral and pelvic limb long bones of Indigenous Naked Neck chickens in Quara District, Northwest Ethiopia. In addition, osteometric measurements were compared between predefined age groups and sexes. We hypothesized that osteometric measurements would differ between the predefined age groups and sexes examined.

## 2 Materials and methods

### 2.1 Study area

The study was conducted in Quara District, Northwest Ethiopia, approximately 1,045 km from Addis Ababa and 325 km from Gondar. The district is located between 11°47′ and 12°21′ N latitude and 35°16′ and 35°47′ E longitude, with elevations ranging from 528 to 654 m above sea level (Fig. 1). Quara District borders Metema, Alefa, and Jawi districts, as well as Benishangul-Gumuz Regional State and the Republic of Sudan. The district covers approximately 7,979.1 km² and comprises two major agroecological zones: Woina Dega (midland), accounting for approximately 2% of the area, and Kola (lowland), representing approximately 98%. The area is characterized by a predominantly hot climate, with annual temperatures ranging from 25 to 44 °C and annual rainfall ranging from 600 to 1,000 mm (Alemu & Alemneh, 2017). Livestock production is an important component of the local agricultural system, with cattle, sheep, goats, poultry, horses, and camels commonly raised.

**Fig. 1:**
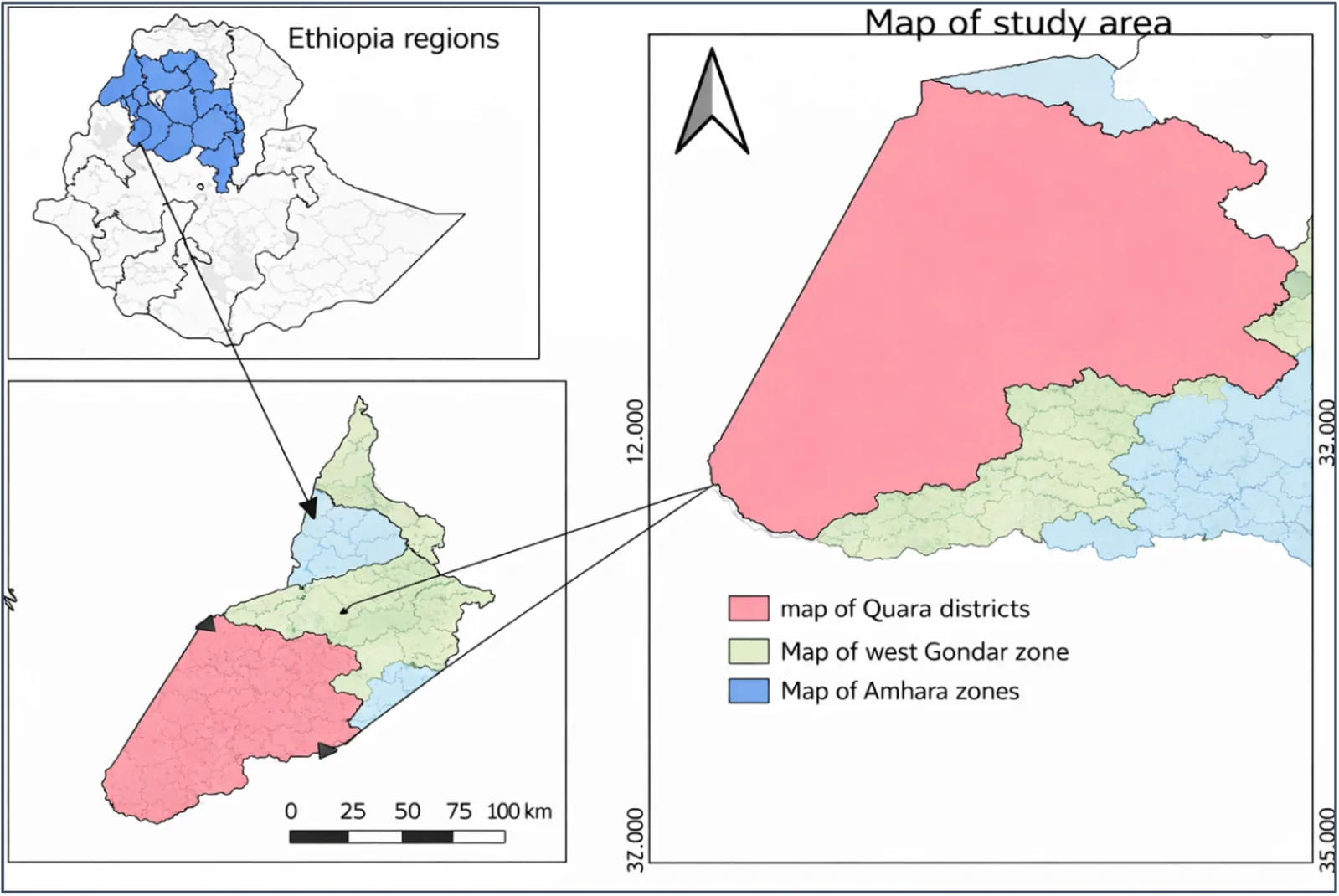
Map of the study area (ArcGIS 10.3.1).

### 2.2 Study animals and husbandry

Sixteen clinically healthy Indigenous Naked Neck chickens (*Gallus gallus domesticus*), comprising eight males and eight females, were included in the study. The birds were categorized into two predefined age groups: 8-month-old chickens (younger group) and 14-month-old chickens (older group), with eight chickens in each group. Each age group comprised four males and four females. Average body weight ranged from 1.6 to 1.7 kg in the younger group and from 1.9 to 2.4 kg in the older group. However, individual body weight records were not available for all chickens and therefore could not be incorporated as a covariate in the statistical analysis. Age was established from owner-provided records and subsequently corroborated by external morphological characteristics, including plumage development, neck morphology, body size, and general physical maturity. Only chickens with moderate body condition, normal posture and gait, intact plumage, and normal behavioral responses were included. Birds were required to be free from visible skeletal deformities, lameness, joint swelling, fractures, or other externally detectable musculoskeletal abnormalities. Chickens showing no clinical signs of systemic illness were considered suitable for inclusion.

Chickens displaying poor body condition, signs of malnutrition, external injuries, lameness, skeletal deformities, joint enlargement, abnormal posture, or apparent clinical illness were excluded. Birds with incomplete plumage, evidence of trauma, or suspected metabolic or infectious diseases were also excluded to minimize potential confounding effects. The selected chickens were transported to the Veterinary Anatomy Laboratory of the College of Veterinary Medicine and Animal Sciences, University of Gondar. Before specimen processing, the birds were maintained under standard husbandry and animal-welfare conditions, with access to feed and clean drinking water and routine monitoring of their general health. Birds were housed in clean and adequately ventilated facilities until specimen processing. Humane euthanasia was performed using 100% carbon dioxide (CO₂) inhalation in a sealed chamber, in accordance with the American Veterinary Medical Association guidelines for the euthanasia of animals (Underwood & Anthony, 2020).

### 2.3 Study design and period

An exploratory comparative cross-sectional study was conducted from February to November 2024 in Quara District, Northwest Ethiopia. The study was designed to describe the gross osteomorphological characteristics and assess osteometric variation of selected long bones in two predefined age groups and both sexes of Indigenous Naked Neck chickens. The study included 16 chickens, equally distributed between the two age groups (n = 8 per group), with balanced sex representation (four males and four females per age group). The humerus, radius, ulna, femur, tibia, and tarsometatarsus were examined using standardized gross anatomical observations and osteometric measurements. Comparisons were performed between the predefined age groups and sexes; however, the study was not designed to evaluate age × sex interactions. The study was intended to generate baseline osteomorphological and osteometric data for the Indigenous Naked Neck chickens examined.

### 2.4 Sample size and sampling method

A total of 16 chickens were selected using purposive sampling based on predefined inclusion and exclusion criteria and the availability of eligible Indigenous Naked Neck chickens in the study area. Because this was an exploratory anatomical study intended to generate baseline data for an under-characterized population, population-specific reference data needed for an a priori power calculation were limited. The sample was therefore structured to provide balanced representation of the two predefined age groups and sexes, comprising eight chickens in each age group and four males and four females within each group. Although average body weight information was available at the group level, individual body weight records were not available for all chickens and therefore could not be incorporated into the statistical analysis. All selected chickens underwent standardized gross osteomorphological examination and osteometric measurement of the humerus, radius, ulna, femur, tibia, and tarsometatarsus.

### 2.5 Sample collection, preparation, and osteomorphological examination

Following euthanasia, the chickens were skinned and soft tissues, including muscles, ligaments, and tendons, were carefully removed within one hour to minimize postmortem tissue degradation. The bones were cleaned by boiling in a 3% sodium hydroxide solution, air-dried at room temperature, degreased in a 30% ammonia solution for seven days, and whitened in a 4% hydrogen peroxide solution for 24 h. The humerus, radius, ulna, femur, tibia, and tarsometatarsus were selected for osteomorphological and osteometric examination. Gross morphological characteristics, including overall shape, curvature, prominent anatomical landmarks, and articular surfaces, were documented using a high-resolution digital camera. Anatomical terminology followed *Nomina Anatomica Avium* and *Nomina Anatomica Veterinaria* (Baumel, 1993; Konig, 2016).

### 2.6 Osteometric measurements

Each bone was weighed in grams using a precision balance. Linear measurements were recorded in millimeters using digital calipers and an osteometric board according to standardized anatomical landmarks. Greatest length was measured as the maximum distance between the most proximal and distal points of each bone. Proximal and distal breadths were measured as the maximum transverse widths across the respective bone extremities, while the smallest diaphyseal breadth was measured at the narrowest portion of the shaft. Additional bone-specific measurements, including breadth of the surgical neck, breadth of the trochlea, and diaphyseal circumference, were recorded where applicable. All measurements were obtained using consistent anatomical landmarks and standardized procedures to improve measurement accuracy and reproducibility. The overall experimental design and study workflow, including animal selection and grouping, sample collection and bone preparation, osteomorphological examination, osteometric measurements, and statistical analysis, are summarized in Fig. 2.

**Fig. 2:**
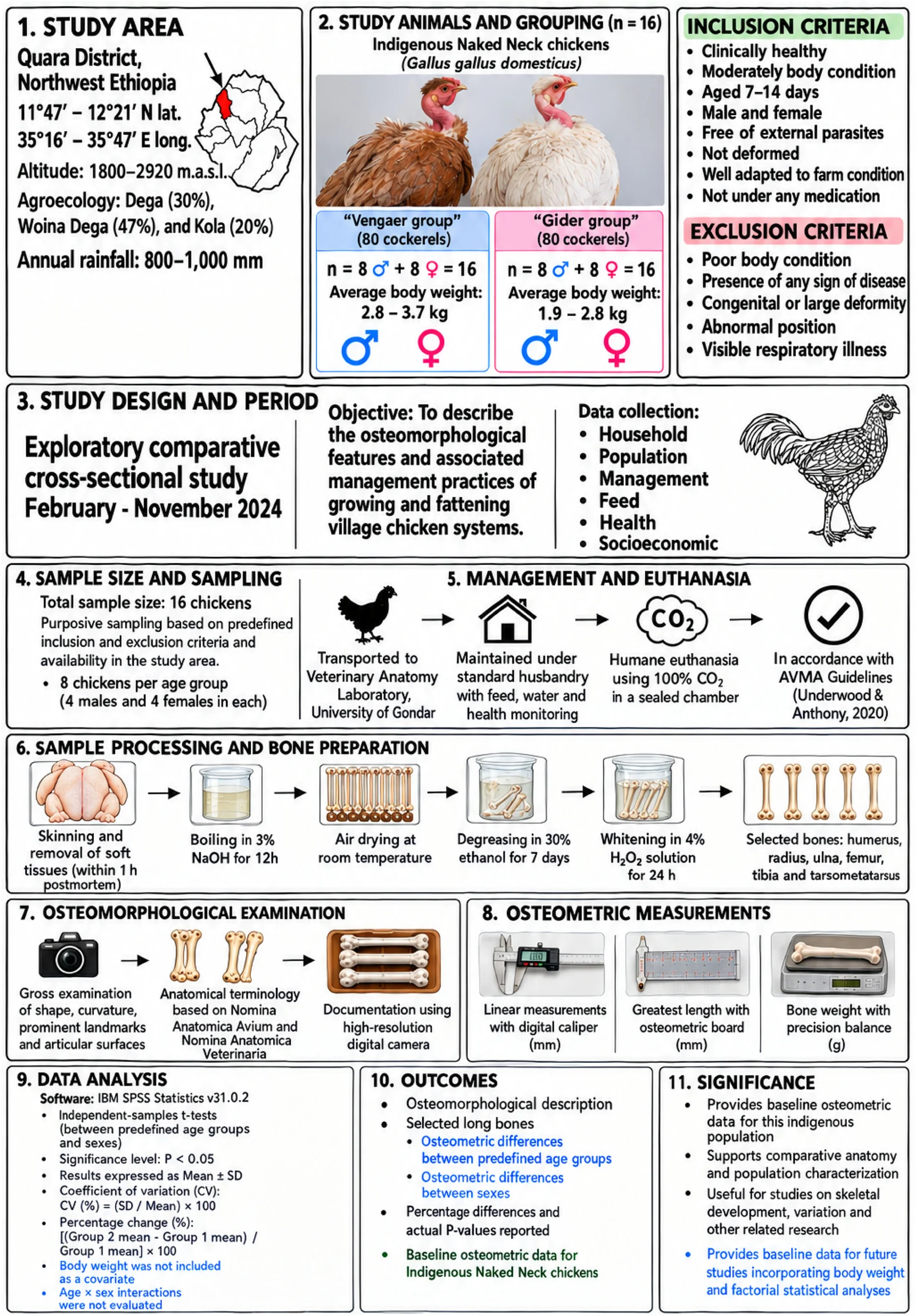
Experimental design and study workflow for osteomorphological and osteometric assessment of long bones in Indigenous Naked Neck chickens.

### 2.7 Data analysis and management

Data were entered, coded, and managed using Microsoft Excel spreadsheets and analyzed using IBM SPSS Statistics version 31.0.2. Independent-samples *t*-tests were used to compare osteometric measurements between the two predefined age groups and between males and females. Statistical significance was set at *P* < 0.05. Results were expressed as mean ± standard deviation (SD). Measurement variability within each group was assessed using the coefficient of variation (CV), calculated as:

$$\text{CV}\left(\%\right)\;=\frac{\;\text{Standard}\;\text{deviation}\;}{\text{Mean}}\times 100$$

The percentage change was also calculated to quantify the magnitude of differences between comparison groups:

$$\text{Percentage}\;\text{change}\;\left(\%\right)\;=\frac{\;\text{Comparison}\;\text{group}\;\text{mean}\;-\;\text{Reference}\;\text{group}\;\text{mean}}{\text{Reference}\;\text{group}\;\text{mean}}\times 100$$

The CV was used to assess relative variability among osteometric parameters measured on different scales. Higher CV values indicated greater relative variability, whereas lower CV values indicated greater measurement consistency within the study groups. Percentage change values were used to describe the direction and magnitude of differences between age and sex categories. Because body weight data were not available, body weight could not be included as a covariate in the statistical analysis. Although the study included age and sex categories, each age-sex subgroup contained only four chickens. Consequently, the study was not specifically powered to evaluate age × sex interaction effects using a factorial GLM. Therefore, independent-samples *t*-tests were used for predefined comparisons between age groups and between sexes, and the results should be interpreted as group-level associations rather than independent estimates of the effects of age, sex, or their interaction.

## 3 Results

### 3.1 Osteomorphology

The humerus exhibited an elliptical head directed medially at the proximal extremity, accompanied by two distinct tubercles separated by a well-defined bicipital groove. The medial tubercle was smaller and less prominent than the lateral tubercle and showed slight cranial curvature (Figs. 3A and 3B). The shaft was cylindrical with mild torsion and displayed a shallow musculospiral groove on the lateral surface. A nutrient foramen was observed on the caudal aspect of the shaft. Distally, the humerus terminated in two condyles, with the medial condyle larger than the lateral. The olecranon fossa was deeper than the radial fossa, and the distal articular surface consisted of a medially positioned grooved trochlea and a laterally broader capitulum.

**Fig. 3:**
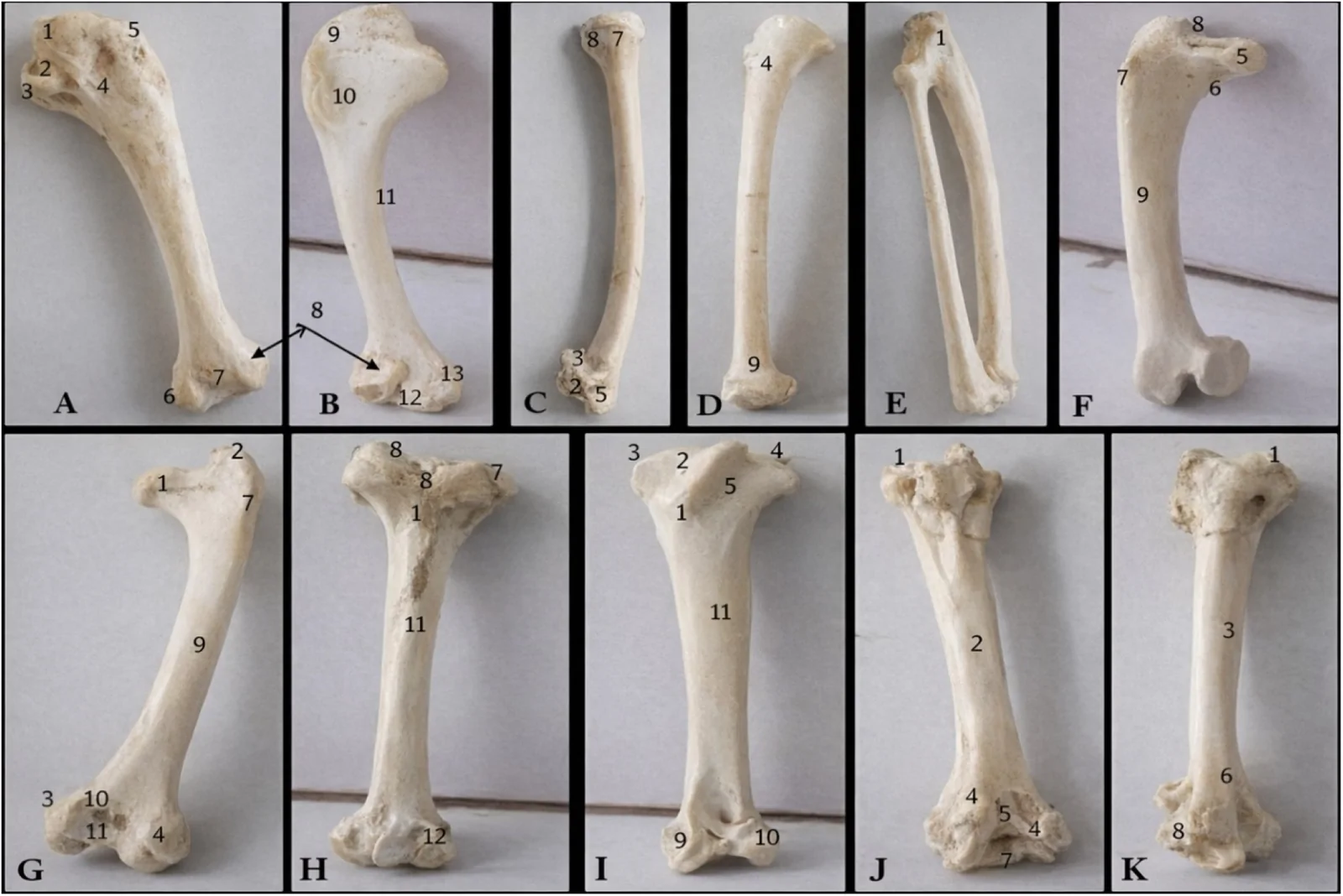
Osteomorphological features of the long bones in Naked Neck chickens. (A) Medial and (B) lateral views of the humerus showing the head (1), capital groove (*sulcus capitalis*) (2), ventral tubercle (3), fossa (4), dorsal tubercle (5), dorsal epicondyle (6), olecranon fossa (7), ventral epicondyle (8), transverse groove (9), shaft (11), ventral condyle (12), and dorsal condyle (13). (C) Lateral view of the ulna and (D) dorsal view of the radius. (E) Medial view of the radius and ulna illustrating the olecranon (1), cotyla (2), radial incisure (3), proximal extremity of the radius (4), proximal extremity of the ulna (5), condyles (6, 7), epicondyle (8), and distal extremity of the radius (9). (F) Cranial and (G) caudal views of the femur showing the caput femoris (1), greater trochanter (2), medial condyle (3), lateral condyle (4), head (5), neck (6), trochanteric crest (7), trochanteric fossa (8), shaft (9), supracondyloid fossa (10), and intercondyloid fossa (11). (H) Medial and (I) dorsal views of the tibia showing the tibial crest (1), tibial tuberosity (2), lateral condyle (3), medial condyle (4), muscular sulcus (5), condylar facets (6, 7), intertuberal fossa (8), lateral malleolus (9), medial malleolus (10), and shaft (11). (J) Ventral and (K) dorsal views of the tarsometatarsus showing the proximal extremity (1), ventral longitudinal groove (2), dorsal longitudinal groove (3), facet for the sesamoid bone (4), anterior groove (5), distal metatarsal canal (6), intercondylar cleft (7), and medial condyle (8).

The ulna, observed in lateral view, was robust and showed a prominent and convex medial styloid projection (Fig. 3C). The radius was positioned cranial to the ulna and exhibited a concave caudal surface and convex cranial surface, resulting in a smaller diameter compared to the ulna (Fig. 3D). It was elongated, craniocaudally flattened, and displayed slight medial curvature. The radius closely adhered to the cranial surface of the ulna along most of its length, except at the regions of the olecranon, cotyla, radial incisure, and the proximal and distal extremities, where distinct separation between the two bones was evident (Fig. 3E).

The femur was generally straight, with gentle curvature apparent in both cranial and caudal views (Fig. 3, Fig. 3). The proximal extremity featured a medially projecting head associated with the trochanteric fossa and a prominent greater trochanter, characterized by a concave medial surface and convex lateral surface. A nutrient foramen was present on the proximal portion of the shaft. Distally, the femur displayed a well-developed trochlea composed of two uneven ridges separated by a longitudinal groove. A deep intercondyloid fossa was observed on the caudal surface between the condyles. The greater and lesser trochanters were connected by a curved trochanteric crest, and a supracondyloid fossa was visible caudally.

The tibia consisted of a cylindrical shaft that expanded proximally and distally (Fig. 3, Fig. 3). The proximal extremity comprised medial and lateral condyles separated by an intertuberal fossa, with distinct articular facets and tibial tuberosities. A tibial spine located between the condyles showed clearly differentiated medial and lateral components. The distal extremity was comparatively narrower and formed articular surfaces for connection with the tarsal bones. A small, separate malleolar bone contributed to the formation of the lateral malleolus.

The tarsometatarsus was elongated with clearly defined dorsal and ventral surfaces (Fig. 3, Fig. 3). The proximal extremity possessed a smooth articular surface and a prominent metatarsal tuberosity. Posterior to the tuberosity, a concave facet and lateral articular areas were present. The shaft was characterized by a narrow dorsal longitudinal groove. The distal extremity displayed an intercondyloid cleft separating the medial and lateral condyles, with the presence of a tarsometatarsal canal and distinct facets for sesamoid bones at both proximal and distal ends.

### 3.2 Osteometric measurements

The osteometric measurements of the long bones of Indigenous Naked Neck chickens are presented in Table 1, Table 2. Older chickens generally exhibited greater mean bone weight and linear dimensions than younger chickens across the six examined long bones. These differences were observed for greatest length, proximal and distal breadths, smallest diaphyseal breadth, and other bone-specific measurements where applicable. The greater dimensions observed in the older group suggest differences between the predefined age groups. However, as body weight was not available for inclusion in the analysis and age × sex interactions were not assessed, these differences should not be interpreted as independent effects of age alone. The percentage differences and corresponding actual *P*-values for individual parameters are presented in Table 1, Table 2.

**Table 1:** Osteometric measurements of long bones by age group in Naked Neck chickens.

**Bone**	**Parameter**	**Mean ± SD**		**P value**	**CV**		**% change**
		**Younger** **(n=8)**	**Older** **(n=8)**		**Younger** **(n=8)**	**Older** **(n=8)**	
Humerus	Weight	4.93 ± 0.50	8.97 ± 0.59	< 0.001	10.14	6.58	81.95%
	GL	75.14 ± 2.88	81.27 ± 2.80	< 0.001	3.83	3.45	8.16%
	BP	22.60 ± 2.03	26.95 ± 1.44	< 0.001	8.98	5.34	19.25%
	BD	16.18 ± 0.70	22.18 ± 0.70	< 0.001	4.33	3.16	37.08%
	SD	6.19 ± 0.49	12.19 ± 0.49	< 0.001	7.92	4.02	96.93%
	BS	6.81 ± 0.58	12.22 ± 2.05	< 0.001	8.52	16.78	79.44%
	BT	14.65 ± 0.67	20.65 ± 0.67	< 0.001	4.57	3.24	40.96%
Radius	Weight	4.19 ± 0.40	8.18 ± 0.47	< 0.001	9.55	5.75	95.23%
	GL	68.25 ± 3.29	74.25 ± 3.29	< 0.001	4.82	4.43	8.79%
	BP	6.73 ± 0.31	12.73 ± 0.31	< 0.001	4.61	2.44	89.15%
	BD	5.29 ± 0.07	11.29 ± 0.07	< 0.001	1.32	0.62	113.42%
	SD	4.67 ± 0.43	8.62 ± 0.40	< 0.001	9.21	4.64	84.58%
Ulna	Weight	4.08 ± 0.57	7.91 ± 0.21	< 0.001	13.97	2.65	93.87%
	GL	76.63 ± 3.25	82.32 ± 3.24	< 0.001	4.24	3.94	7.43%
	BP	12.21 ± 0.79	18.21 ± 0.79	< 0.001	6.47	4.34	49.14%
	BD	11.05 ± 1.11	15.18 ± 0.17	< 0.001	10.05	1.12	37.38%
	SD	5.63 ± 0.87	10.35 ± 0.18	< 0.001	15.45	1.74	83.84%
Femur	Weight	6.56 ± 0.79	11.73 ± 1.06	< 0.001	12.04	9.04	78.81%
	GL	85.72 ± 4.15	93.18 ± 3.54	< 0.001	4.84	3.80	8.70%
	BP	15.08 ± 1.98	21.18 ± 1.75	< 0.001	13.13	8.26	40.45%
	BD	16.94 ± 1.26	22.94 ± 1.26	< 0.001	7.44	5.49	35.42%
	SD	6.67 ± 0.01	12.67 ± 0.01	< 0.001	0.15	0.08	89.96%
	CD	22.61 ± 0.70	26.98 ± 0.67	< 0.001	3.10	2.50	19.33%
Tibia	Weight	7.04 ± 1.46	13.05 ± 1.46	< 0.001	20.74	11.19	85.37%
	GL	127.06 ± 5.12	134.95 ± 4.42	< 0.001	4.03	3.28	6.21%
	BP	22.76 ± 1.77	28.76 ± 1.76	< 0.001	7.78	6.12	26.36%
	BD	11.92 ± 0.43	17.92 ± 0.43	< 0.001	3.61	2.40	50.34%
	SD	5.69 ± 0.10	11.69 ± 0.10	< 0.001	1.76	0.86	105.45%
Tarsometatarsus	Weight	4.34 ± 1.29	10.09 ± 1.15	< 0.001	29.72	11.40	132.49%
	GL	88.56 ± 5.28	96.36 ± 4.47	< 0.001	5.96	4.64	8.81%
	BP	14.45 ± 1.20	20.45 ± 1.20	< 0.001	8.30	5.87	41.52%
	BD	14.93 ± 0.87	20.93 ± 0.87	< 0.001	5.83	4.16	40.19%
	SD	4.52 ± 0.24	10.52 ± 0.24	< 0.001	5.31	2.28	132.74%
	CD	15.96 ± 1.49	20.18 ± 0.75	< 0.001	9.34	3.72	26.44%

Note: Values are expressed as Mean ± Standard Deviation (SD), measured in millimeters (mm), except for bone weight, which is expressed in grams (g). CV (%) denotes the coefficient of variation (SD/Mean × 100). % change was calculated as [(Older mean − Younger mean)/Younger mean] × 100. Differences were considered statistically significant at *P* < 0.05. Abbreviations: GL = Greatest Length; BP = Breadth of Proximal End; BD = Breadth of Distal End; SD = Smallest Breadth of Diaphysis; BS = Breadth of Surgical Neck; BT = Breadth of Trochlea; CD = Circumference of Diaphysis; N = number of individuals examined.

**Table 2:** Osteometric measurements of long bones by sex in Naked Neck chickens.

**Bone**	**Parameter**	**Mean ± SD**		**P value**	**CV**		**% change**
		**Female** **(n=8)**	**Male** **(n=8)**		**Female** **(n=8)**	**Male (n=8)**	
Humerus	Weight	3.845 ± 0.403	8.576 ± 0.559	< 0.001	10.48	6.52	123.04%
	GL	72.843 ± 0.776	82.108 ± 1.265	< 0.001	1.07	1.54	12.72%
	BP	19.311 ± 0.203	23.270 ± 0.552	< 0.001	1.05	2.37	20.50%
	BD	15.143 ± 0.070	20.143 ± 0.070	< 0.001	0.46	0.35	33.02%
	SD	5.651 ± 0.112	10.651 ± 0.112	< 0.001	1.98	1.05	88.48%
	BS	6.252 ± 0.192	11.252 ± 0.192	< 0.001	3.07	1.71	79.97%
	BT	14.293 ± 0.461	19.293 ± 0.461	< 0.001	3.23	2.39	34.98%
Radius	Weight	3.941 ± 0.386	7.539 ± 0.834	< 0.001	9.79	11.06	91.30%
	GL	65.048 ± 1.079	73.598 ± 1.151	< 0.001	1.66	1.56	13.14%
	BP	6.597 ± 0.294	11.597 ± 0.294	< 0.001	4.46	2.54	75.79%
	BD	5.499 ± 0.103	10.499 ± 0.103	< 0.001	1.87	0.98	90.93%
	SD	4.721 ± 0.542	8.663 ± 0.768	< 0.001	11.48	8.87	83.50%
Ulna	Weight	4.079 ± 0.541	7.652 ± 0.030	< 0.001	13.26	0.39	87.59%
	GL	70.902 ± 0.904	81.185 ± 1.200	< 0.001	1.27	1.48	14.50%
	BP	11.619 ± 0.400	16.619 ± 0.400	< 0.001	3.44	2.41	43.03%
	BD	10.371 ± 0.821	14.105 ± 0.664	< 0.001	7.92	4.71	36.00%
	SD	4.153 ± 0.039	9.153 ± 0.039	< 0.001	0.94	0.43	120.39%
Femur	Weight	4.526 ± 0.212	10.526 ± 0.212	< 0.001	4.68	2.01	132.57%
	GL	82.212 ± 1.802	92.407 ± 1.437	< 0.001	2.19	1.56	12.40%
	BP	14.780 ± 2.073	19.780 ± 2.073	< 0.001	14.03	10.48	33.83%
	BD	16.124 ± 0.753	21.124 ± 0.753	< 0.001	4.67	3.56	31.01%
	SD	6.683 ± 0.028	11.683 ± 0.028	< 0.001	0.42	0.24	74.82%
	CD	21.765 ± 1.321	26.235 ± 1.071	< 0.001	6.07	4.08	20.54%
Tibia	Weight	6.442 ± 0.628	11.567 ± 0.441	< 0.001	9.75	3.81	79.56%
	GL	120.593 ± 0.457	134.743 ± 1.441	< 0.001	0.38	1.07	11.73%
	BP	20.917 ± 0.480	25.862 ± 0.399	< 0.001	2.29	1.54	23.64%
	BD	11.804 ± 0.388	16.804 ± 0.388	< 0.001	3.29	2.31	42.36%
	SD	5.831 ± 0.237	10.831 ± 0.237	< 0.001	4.06	2.19	85.75%
Tarsometatarsus	Weight	5.024 ± 0.629	8.899 ± 0.285	< 0.001	12.52	3.20	77.13%
	GL	81.743 ± 0.337	96.303 ± 1.135	< 0.001	0.41	1.18	17.81%
	BP	13.457 ± 0.531	18.457 ± 0.531	< 0.001	3.95	2.88	37.16%
	BD	14.109 ± 0.299	19.109 ± 0.299	< 0.001	2.12	1.56	35.44%
	SD	4.263 ± 0.060	9.263 ± 0.060	< 0.001	1.41	0.65	117.29%
	CD	14.866 ± 0.949	19.030 ± 0.790	< 0.001	6.38	4.15	28.01%

Note: Values are expressed as mean ± standard deviation (SD), measured in millimeters (mm), except for bone weight, which is expressed in grams (g). CV (%) denotes the coefficient of variation, calculated as (SD/Mean × 100). Percentage change (%) was calculated as [(Male mean − Female mean) / Female mean] × 100. Differences were considered statistically significant at *P* < 0.05. Abbreviations: GL = Greatest Length; BP = Breadth of Proximal End; BD = Breadth of Distal End; SD = Smallest Breadth of Diaphysis; BS = Breadth of Surgical Neck; BT = Breadth of Trochlea; CD = Circumference of Diaphysis; *n* = number of individuals examined.

Male chickens also generally exhibited greater mean bone weight and linear dimensions than females across the examined long bones. This pattern indicates differences between male and female chickens within the studied population. However, the contribution of overall body size and potential age × sex interactions could not be evaluated. The percentage differences and actual *P*-values for individual comparisons are provided in the corresponding tables. The coefficients of variation (CV) demonstrated differences in relative variability among the measured parameters. Bone weight generally showed greater relative variability than linear measurements, whereas measurements with lower CV values showed greater consistency within the examined groups.

## 4 Discussion

Bone growth and morphology in poultry are shaped by multiple interacting factors, including mineral availability, genetic background, physiological regulation, nutrition, and mechanical loading. Calcium and phosphorus constitute the primary mineral components of the skeletal matrix and play essential roles in bone mass, mineralization, and linear growth, although their bioavailability may vary depending on diet and environmental conditions (Cashman, 2002; Wawrzyniak & Suliburska, 2021). Nutritional homeostasis of these minerals is tightly regulated through endocrine mechanisms that influence both skeletal growth and maintenance (Rigo et al., 2012; Tamamura & Yamaguchi, 2012). These factors provide a biological basis for interpreting the age- and sex-related differences observed in the present study.

In the present study, the osteomorphological features of the examined long bones generally conformed to established patterns of avian skeletal anatomy. The humerus exhibited a rounded, medially oriented head, clearly defined tubercles, a distinct bicipital groove, and differentiated distal condyles. These structures provide surfaces for muscle attachment and articulation and therefore contribute to movement and stabilization of the wing. The observed configuration was consistent with descriptions of domestic chickens and other avian taxa (Wise, 1970; Cubo et al., 1999; Serrano et al., 2020). Variation in the prominence of the humeral tubercles among bird species may be related to differences in muscle development and locomotor demands, as suggested by comparative studies of avian skeletal morphology (Mayr, 2010; Kondoh et al., 2022). The less prominent tubercles observed in the present chickens compared with those described in larger-bodied birds such as geese may therefore reflect differences in body size and associated muscular and functional requirements (Serrano et al., 2020; Doğan & Takcı, 2021).

The radius and ulna displayed close apposition along most of their shafts, with separation around the proximal and distal extremities. This arrangement likely provides structural stability while maintaining coordinated movement of the forearm. The greater robustness of the ulna relative to the radius may also contribute to the mechanical support and muscle attachment of the forelimb. These observations agree with previous osteometric and anatomical descriptions of indigenous chickens and guinea fowl (Visto et al., 2018; Abdulrahman & Yusuf, 2021). Comparable curvature and distal features have also been described in mammalian ulnae, suggesting that some structural characteristics are associated with common biomechanical requirements of limb function across vertebrates (Sharma et al., 2011).

The femur showed moderate shaft curvature, a prominent greater trochanter, and a well-developed distal trochlea. These features provide attachment sites for muscles and contribute to stabilization and transmission of forces through the pelvic limb during locomotion and weight bearing. The observed morphology is consistent with descriptions of femoral structure in birds and other weight-bearing vertebrates (Kielan-Jaworowska & Gambaryan, 1994; Nomir et al., 2024). The similarity of these structural features across taxa suggests functional convergence associated with locomotion, posture, and transmission of mechanical forces.

The tibia was the longest and heaviest long bone examined in the present study and possessed expanded proximal and distal extremities with a cylindrical diaphysis. The relatively large size and robust structure of the tibia are functionally appropriate because this bone forms a major component of the weight-bearing pelvic limb and transmits forces generated during standing and locomotion. Similar structural characteristics have been reported in large avian species such as ostriches (Kassem et al., 2023). Comparable morphofunctional adaptations have also been documented in the pelvic limbs of terrestrial mammals, including cervids and bovids, where robust long bones contribute to body support and locomotor stability (Rajani et al., 2013; Bharti & Singh, 2020).

The tarsometatarsus was elongated and exhibited distinct condyles, longitudinal grooves, and well-defined articular facets. These features provide rigidity and facilitate the transmission and distribution of forces between the proximal limb and digits during standing and locomotion. The observed morphology therefore reflects the functional specialization of the avian distal limb for bipedal locomotion. Comparable structural patterns have been reported in distal limb elements of ungulates, where articular and shaft morphology similarly contributes to limb stabilization and load distribution (Choudhary & Singh, 2016; Ekeolu et al., 2016).

The osteometric findings further demonstrated that the long bones of Indigenous Naked Neck chickens were smaller than those reported for larger poultry species such as geese. This difference is expected because skeletal dimensions generally scale with overall body size and the mechanical demands imposed on the skeleton. The lower humeral length and breadth observed in the present chickens compared with larger birds are consistent with previously reported measurements in larger avian species (Jáuregui et al., 2015; Serrano et al., 2020). Such interspecific differences in skeletal size are more likely to reflect variation in body mass, growth rate, and functional requirements than pathological changes (Reis et al., 2011).

Older chickens consistently exhibited greater bone weight and osteometric dimensions than younger chickens across the examined long bones. This pattern can be explained by continued skeletal growth, involving both longitudinal enlargement and increases in bone mass and mineral deposition as chickens mature. The percentage changes further indicated that the magnitude of age-related differences varied among parameters. Bone weight and some breadth measurements showed larger percentage changes than greatest length, suggesting that skeletal development involved not only elongation but also substantial increases in transverse dimensions and bone mass. These observations are consistent with previous reports describing larger skeletal dimensions in older poultry. However, because body weight was not available and age × sex interactions were not evaluated, the observed differences should be interpreted as associations rather than independent age effects (Zein-el-dein et al., 1981; Rose et al., 1996; Behera et al., 2021).

Differences between sexes were observed, with males generally exhibiting greater bone weight and osteometric dimensions than females. This pattern indicates sexual dimorphism in skeletal development within the studied Naked Neck population. The larger measurements in males may be related to differences in overall body size, growth rate, muscle development, and hormonal influences on skeletal growth. The percentage changes showed that sex-related differences were particularly pronounced for bone weight and some breadth measurements, whereas differences in greatest length were generally smaller. This suggests that sexual dimorphism may be expressed more strongly through skeletal mass and transverse development than through longitudinal dimensions. Similar sex-related differences have been reported in Naked Neck chickens and other poultry strains (Zein-el-dein et al., 1981; Rose et al., 1996; Kranioti & Michalodimitrakis, 2009).

The magnitude of skeletal variation may also be influenced by environmental and management factors. Differences in management systems and nutritional regimes can affect growth, mineral deposition, and skeletal development, potentially contributing to variability within a chicken population. Such effects have been reported specifically in Naked Neck chickens (Bughio et al., 2020; Markos et al., 2024). Therefore, the age- and sex-related differences observed in the present study may reflect not only intrinsic biological variation but also the combined influence of nutritional and management conditions.

Variability in osteometric parameters differed among the measurements examined. Bone weight generally showed relatively higher coefficients of variation, whereas several linear measurements, particularly longitudinal dimensions, exhibited lower CV values. Greater variation in bone weight may result from individual differences in body size, growth rate, nutritional status, and mineral deposition, whereas linear dimensions may be more developmentally constrained (Mabelebele et al., 2017; Behera et al., 2021). The observed variation among different bone regions may also reflect differences in growth patterns and mechanical loading. Previous studies of bone mechanical properties and comparative osteology have similarly demonstrated variation in structural and mechanical characteristics among different regions of bones (Currey et al., 2006; Ekeolu et al., 2016). However, the CV values in the present study should be interpreted as measures of relative dispersion within the sampled population rather than direct measures of bone strength or mechanical performance. Nevertheless, because body weight was not recorded and included as a covariate, part of the observed differences may reflect variation in overall body size rather than sex-specific skeletal effects alone.

## 5 Limitations

This study has several limitations. The relatively small sample size may have reduced statistical power. Only two age groups from a single geographic population were examined, which may limit the generalizability of the findings. Although average body weight information was available at the group level, individual body weight records were not available for all chickens and therefore could not be incorporated as a covariate in the statistical analysis. Consequently, some differences observed between age groups and sexes may partly reflect variation in overall body size. Furthermore, age × sex interaction effects were not evaluated because of the limited sample size within age-sex subgroups. Therefore, the observed associations should not be interpreted as independent effects of age or sex.

## 6 Conclusion

The long bones of Indigenous Naked Neck chickens from Quara District exhibited osteomorphological features consistent with established avian skeletal anatomy. Osteometric measurements differed between the predefined age groups and sexes, with older chickens and males generally showing greater bone weight and linear dimensions. However, because individual body weight data were not available for inclusion as a covariate and age × sex interactions were not evaluated, these findings should be interpreted as observed group differences rather than independent effects of age or sex. The study provides baseline osteological data for Indigenous Naked Neck chickens and may serve as a reference for comparative anatomical, developmental, and population-based investigations. Future studies should include larger and more diverse populations, individual body weight measurements, and factorial statistical approaches to better characterize skeletal variation and development.

## Funding

No funding was received for this study.

## Ethics declaration

This study was conducted in accordance with the ARRIVE (Animal Research: Reporting of In Vivo Experiments) guidelines. This study was approved by the Ethical clearance was obtained from the Institutional Animal Care and Use Committee (IACUC) of the University of Gondar, College of Veterinary Medicine and Animal Sciences (Ref. No. CVMASc/UoG/RERC/13/03/2024; approved April 1, 2024). Informed consent was obtained from all chicken owners prior to sample collection. All procedures followed established animal welfare guidelines. Humane euthanasia was performed using carbon dioxide (CO₂) inhalation.

## CRediT authorship contribution statement

**Zerihun Getie Wassie:** Writing – review & editing, Writing – original draft, Visualization, Validation, Software, Resources, Methodology, Investigation, Formal analysis, Data curation, Conceptualization. **Bemrew Admassu Mengistu:** Visualization, Validation, Supervision, Methodology. **Gashaw Getaneh Dagnaw:** Visualization, Validation, Supervision, Methodology. **Saleamlak Abebe Bihonegn:** Writing – review & editing, Visualization, Validation, Resources. **Abdo Megra Geda:** Writing – review & editing, Visualization, Validation, Resources. **Assaye Wollelie Fentie:** Writing – review & editing, Visualization, Validation, Resources. **Teketay Bayleyegn Derso:** Writing – review & editing, Visualization, Validation, Resources. **Kefale Ambachew Shiferaw:** Writing – review & editing, Visualization, Validation, Resources, Methodology. **Abraham Belete Temesgen:** Writing – review & editing, Writing – original draft, Visualization, Validation, Supervision, Resources, Methodology, Data curation, Conceptualization.

## Declaration of competing interest

The authors declare that they have no known competing financial interests or personal relationships that could have appeared to influence the work reported in this paper.

## Data Availability

The data supporting the findings of this study are available from the corresponding author upon reasonable request.

## References

[bib0001] Abdulrahman B., Yusuf A. (2021). Comparative osteometric study of some selected bones of local domestic turkey and guinea fowl. Insights Vet Sci.

[bib0002] Alemu G., Alemneh T. (2017). The prevalence of bovine trypanosomiasis in Quara Woreda, North Western of Ethiopia. Journal of Veterinary Medicine Research.

[bib0003] Baumel J.J. (1993). Handbook of avian anatomy: nomina anatomica avium. Publications of the Nuttall Ornithological Club (USA). no. 23.

[bib0004] Behera M., Sathapathy S., Mandal A.K. (2021). Gross morphometrical changes in leg bones of post-hatch male broiler chickens with special reference to growth plate cartilage. Exploratory Animal and Medical Research.

[bib0005] Bharti, S. K., & Singh, I. (2020). Morphometrical studies of tibia, fibula and lateral malleolus of the blue bull (Boselephus tragocamelus).

[bib0006] Bughio E., Hussain J., Mahmud A., Khalique A. (2020). Effect of production systems and dietary interventions on growth performance, morphometrics, physiological response and behaviour of the naked neck chickens. Brazilian Journal of Poultry Science.

[bib0007] Cashman K. (2002). Calcium intake, calcium bioavailability and bone health. British journal of Nutrition.

[bib0008] Chaurasia, D., Ingole, S. P., Deshmukh, S. K., Netty, S., & Mandavi, S. (2018). Comparative gross morphometrical study on appendicular skeleton of duck and fowl.

[bib0009] Choudhary O.P., Singh I. (2016). Morphological and Radiographic Studies on the Skull of Indian Blackbuck (Antilope cervicapra). International Journal of Morphology.

[bib0010] Cubo J., Menten L., Casinos A. (1999). Sagittal long bone curvature in birds. Annales des Sciences Naturelles-Zoologie et Biologie Animale.

[bib0011] Currey J.D., Pitchford J.W., Baxter P.D. (2006). Variability of the mechanical properties of bone, and its evolutionary consequences. Journal of the Royal Society Interface.

[bib0012] Doğan G.K., Takcı İ. (2021). A macroanatomic, morphometric and comparative investigation on skeletal system of the geese growing in Kars region II; Skeleton appendiculare. Black Sea Journal of Health Science.

[bib0013] Ekeolu, O. K., Usende, I. L., Adejumobi, O. A., Azeez, A. I., & Orolu-Adedeji, M. O. (2016). Comparative morphometric study of the right and left pectoral and pelvic bones of cattle egret (Bubulcus ibis).

[bib0014] Frey R., Albert R., Krone O., Lierz M. (2001). Osteopathy of the pectoral and pelvic limbs including pentadactyly in a young Kestrel (Falco t. tinnunculus). Journal für Ornithologie.

[bib0015] Getu A., Alemayehu K., Wuletaw Z. (2014). Phenotypic characterization of indigenous chicken ecotypes in the north Gondar zone, Ethiopia. Animal Genetic Resources/Resources génétiques animales/Recursos genéticos animales.

[bib0016] Jáuregui R., Flores H., Vasquez L., Oliva M.J. (2015). Caracterización morfométrica de la gallina de cuello desnudo Gallus domesticus nudecollis en la región ch'ortí de Chiquimula, Guatemala. Ciencia, Tecnología y Salud.

[bib0017] Kassem M.A.M., Tahon R.R., Khalil K.M., El-Ayat M.A. (2023). Morphometric studies on the appendicular bony skeleton of the ostriches (Struthio camelus). BMC Veterinary Research.

[bib0018] Khan K., Kilimci F.S., Kara M. (2021). Biomechanical tests: applications and their reliability for the prediction of bone strength in broiler chicken. Veterinary Journal of Mehmet Akif Ersoy University.

[bib0019] Kielan-Jaworowska, Z., & Gambaryan, P. P. (1994). Postcranial anatomy and habits of Asian multituberculate mammals. In *Postcranial anatomy and habits of Asian multituberculate mammals* (pp. 1-92).

[bib0020] Kigir E.S., Omowumi L.K., Onwuama K.T., AZ J., Salami S.O. (2022). Gross anatomical investigation on the fore limb skeleton of the adult helmeted Guinea fowl (Numida meleagridis). Journal of Sustainable Veterinary & Allied Sciences.

[bib0021] Kondoh D., Ono R., Yanagawa M., Goto T. (2022). Morphological variations of caudal skeleton between three chicken breeds. Journal of Veterinary Medical Science.

[bib0022] Konig H.E. (2016).

[bib0023] Kranioti E.F., Michalodimitrakis M. (2009). Sexual dimorphism of the humerus in contemporary Cretans—a population-specific study and a review of the literature. Journal of forensic sciences.

[bib0024] Mabelebele, M., Norris, D., Siwendu, N. A., Ng'ambi, J., Alabi, O. J., & Mbajiorgu, C. (2017). Bone morphometric parameters of the tibia and femur of indigenous and broiler chickens reared intensively.

[bib0025] Markos S., Belay B., Dessie T. (2024). Morphometric differentiation of three chicken ecotypes of Ethiopia using multivariate analysis. PLoS One.

[bib0026] Mayr G. (2010). A new avian species with tubercle-bearing cervical vertebrae from the Middle Eocene of Messel (Germany). Records of the Australian Museum.

[bib0027] Nomir A., Sharaby A.E., Abumandour M.M. (2024). Anatomical studies on the PES region of Zebu cattle (Bos Taurus indicus) with special references to 3D computed tomography imaging technique. BMC veterinary research.

[bib0028] Rajani C.V., Chandrasekhar L., Chandy G., Chungath J.J. (2013). Anatomical studies on the bones of the pelvic limb in Indian muntjac (Muntiacus muntjak). Tamilnadu J. Vet. Anim. Sci.

[bib0029] Reis, D. D. C., Torres, R. D. A., Barbosa, A. D. A., Rodrigues, C. D. S., & Moraes, G. D. (2011). Effects of lineage and sex on geometrical and biomechanical properties of broiler chickens tibias.

[bib0030] Rigo J., Pieltain C., Viellevoye R., Bagnoli F. (2012). Neonatology: A practical approach to neonatal diseases.

[bib0031] Rose N., Constantin P., Leterrier C. (1996). Sex differences in bone growth of broiler chickens. Growth, development, and aging: GDA.

[bib0032] Serrano F.J., Costa-Pérez M., Navalón G., Martín-Serra A. (2020). Morphological disparity of the humerus in modern birds. Diversity.

[bib0033] Sharma A., Kumar A., Singh P. (2011). Anatomical study of the distal end of cadaveric human ulnae: a clinical consideration for the management of distal radioulnar joint injuries. Singapore medical journal.

[bib0034] Tamamura Y., Yamaguchi A. (2012). Bone and tooth in calcium and phosphate metabolism. Clinical Calcium.

[bib0035] Tatara M.R., Krupski W., Majcher P., Studzinski T. (2005). Long-term denervation of the humerus in turkeys as an experimental model for osteopenia. Poultry science.

[bib0036] Thomas R., Sadler P., Cooper J. (2016). Developmental osteology of cross-bred red junglefowl (Gallus gallus L. 1758) and the implications for ageing chickens from archaeological sites. International Journal of Osteoarchaeology.

[bib0037] Underwood W., Anthony R. (2020). AVMA guidelines for the euthanasia of animals: 2020 edition. Retrieved on March.

[bib0038] Visto W.A., Kalhoro I.B., Shah M.G., Laghari M.F., Tarique I., Ahmed N., Liu Y. (2018). Comparative osteometric difference in ulna and radius of commercial layer and desi chicken. Journal of Agriculture and Veterinary Science.

[bib0039] Vitorović D., Pavlovski Z., Škrbić Z., Lukić M., Petričević V., Adamović I.D. (2009). Morphometric and mechanical characteristics of leg bones in autochtonous naked neck breeds of chickens in Serbia. Biotechnology in Animal Husbandry.

[bib0040] Wawrzyniak N., Suliburska J. (2021). Nutritional and health factors affecting the bioavailability of calcium: a narrative review. Nutrition Reviews.

[bib0041] Wise D.R. (1970). Comparisons of the skeletal systems of growing broiler and laying strain chickens. British Poultry Science.

[bib0042] Zeinel-el-dein A., Zeiny M., Ayoub H. (1981). Carcass measurements of naked neck and normal chicks. Annales de Génétique et de Sélection animale.

